# Different mutations of the human *c-mpl* gene indicate distinct haematopoietic diseases

**DOI:** 10.1186/1756-8722-6-11

**Published:** 2013-01-25

**Authors:** Xin He, Zhigang Chen, Yangyan Jiang, Xi Qiu, Xiaoying Zhao

**Affiliations:** 1 Department of Hematology, the Second Affiliated Hospital, Zhejiang University School of Medicine, Hangzhou, 310009, China; 2 Department of Oncology, the Second Affiliated Hospital, Zhejiang University School of Medicine, Hangzhou, 310009, China; 3 UItrasonic Diagnosis Deparment, the Second Affiliated Hospital, Zhejiang University School of Medicine, Hangzhou, 310009, China

**Keywords:** *MPL*, Mutations, Mechanism, Individual therapy, Haematopoietic diseases

## Abstract

The human c-mpl gene (*MPL*) plays an important role in the development of megakaryocytes and platelets as well as the self-renewal of haematopoietic stem cells. However, numerous *MPL* mutations have been identified in haematopoietic diseases. These mutations alter the normal regulatory mechanisms and lead to autonomous activation or signalling deficiencies. In this review, we summarise 59 different *MPL* mutations and classify these mutations into four different groups according to the associated diseases and mutation rates. Using this classification, we clearly distinguish four diverse types of *MPL* mutations and obtain a deep understand of their clinical significance. This will prove to be useful for both disease diagnosis and the design of individual therapy regimens based on the type of *MPL* mutations.

## Introduction

The human c-mpl gene (*MPL)*, a cellular homologue of the oncogene v-mpl, belongs to the haematopoietic receptor superfamily [1,2]. The protein encoded by *MPL*, CD110, is a 635-amino acid protein consisting of four functional domains: the putative signal peptide, the extracellular domain, the transmembrane domain and the intracellular domain. These domains correspond to exon 1, exons 2-9, exon 10 and exons 11-12, respectively [3].

In the absence of the CD110 ligand, thrombopoietin (TPO), the extracellular domain, transmembrane domain and an amphipathic motif, KWQFP, localised at the transmembrane-cytoplasmic junction, retain CD110 in the inactive state [4-6]. When TPO binds to CD110, CD110 is dimerised and the tyrosine kinases JAK2 and TYK2 become activated, which in turn triggers the activation of downstream positive signalling pathways, including JAK-STAT/Crkl, SHP2-Bad, SHC-MAPK, and PLC-PKC [7-19]. It has been suggested that CD110 and TPO play an important role in the development of megakaryocytes and platelets as well as the self-renewal of haematopoietic stem cells [3,20,21].

**Figure 1:**
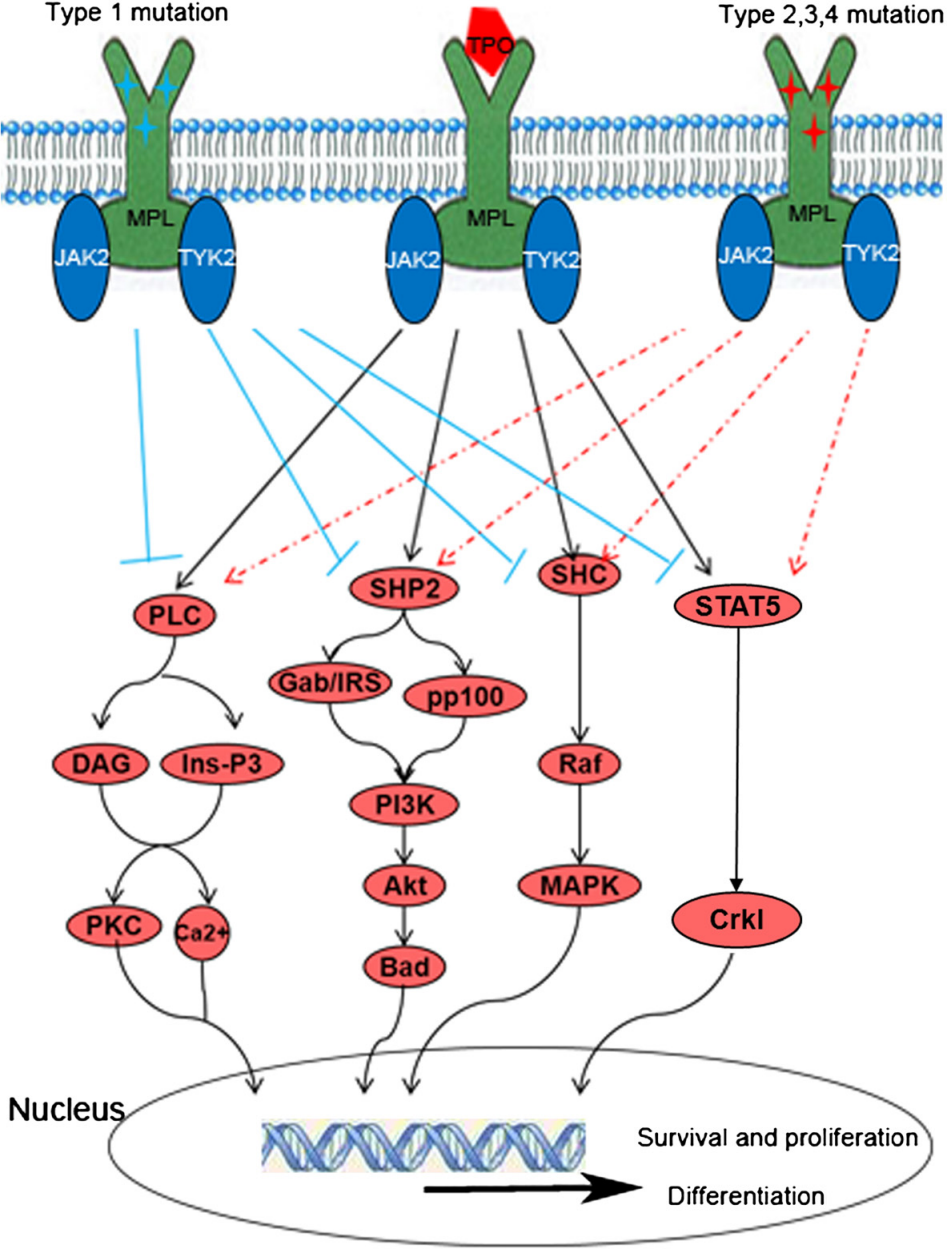
**A schematic summary of the mechanism that different mutations of human c-mpl gene (MPL) bring about distinct biological effects. Type 1 mutation:** MPL mutations found in CAMT. **Type 2 mutation:** MPL-S505N、K39N、 P106L mutations found in HT. **Type 3 mutation:** MPL-W515L and W515K mutations found in MPNs, RARS-t and AML. **Type 4 mutation:** MPL-W515A, W515R, W515-P518delinsKT, Y591D, A519T, L510P, A506T, T487A, Y252H, S204P, S204F, IVS 11/12 and IVS 10/11.

However, various *MPL* mutations alter the normal regulatory mechanisms; some may cause a complete or partial loss of CD110 function, thus leading to thrombocytopenia, while other types of mutations result in the autonomous activation of CD110 and the development of thrombocytosis (Summarised in Figure 1).

### Several *MPL* mutations have been identified in association with haematopoietic diseases

Since the first two heterozygous *MPL* point mutations were identified in congenital amegakaryocytic thrombocytopenia (CAMT) in 1999, mutations in hereditary thrombocythaemia (HT), myeloproliferative neoplasms (MPNs), refractory anaemia with ringed sideroblasts associated with marked thrombocytosis (RARS-t) and acute myeloid leukaemia (AML) have been identified [22]. CAMT is a rare inherited disorder that presents with thrombocytopenia at birth, which eventually progresses into pancytopenia without physical malformations. *MPL* mutations associated with this disease are autosomal, recessive germline mutations, which play a decisive role in the course of the disease [23,24]. According to the data collected by Ballmaier et al., we calculated the mutation rate of the *MPL* gene to reach as high as 91.8% (56 out of 61 CAMT patients for whom *MPL* sequencing revealed *MPL* mutations); no mutations other than those found in the *MPL* gene have been identified in CAMT patients [25]. HT, a hereditary disease of thrombocythaemia, is the second disease that was shown to be associated with *MPL* mutations. The *MPL* mutations detected in HT patients, S505N, K39N and P106L, are also germline mutations. These mutations are inherited in a number of ways: autosomal dominant inheritance, autosomal dominant inheritance with incomplete penetrance and autosomal recessive inheritance with possible mild heterozygote manifestation, respectively. Various somatic mutations, such as *MPL*-W515L, W515K, W515A, W515R, and W515-P518delinsKT, have also been detected in MPN samples, such as polycythaemia vera (PV), primary myelofibrosis (PMF) and essential thrombocythaemia (ET), which manifest as the overproduction of one or more myeloid lineages.

### Classification of *MPL* mutations

We reviewed relevant papers published from 1999 to 2012 and found that 59 different *MPL* mutations have been reported. According to the frequency of *MPL* mutations and the associated clinical conditions, such as inheritance pattern, diagnosis, clinical manifestation and laboratory results, we categorised these mutations into four different groups as follows (summarised in Table 1):

Type1. *MPL* mutations found in CAMT;

Type 2. *MPL* - S505N, K39N and P106L mutations found in HT;

Type 3. *MPL* - W515L and W515K mutations found in MPNs, RARS-t and AML;

Type 4. Other *MPL* mutations: *MPL*-W515A, W515R, W515-P518delinsKT, Y591D, A519T, L510P, A506T, T487A, Y252H, S204P, S204F, IVS 11/12 and IVS 10/11.

**Table 1:** Classfication and clinical features of MPL mutations indentified in hematopoietic diseases

**Mutation type**	**Mutation and Inheritance pattern**	**Effect**	**clinical manifestation**	**Disease (mutation rate)**	**Reference**
Type 1 41 different MPL mutations in CAMT	Autosomal recessive Germline mutation	Completely lose or significantly reduce MPL function	Thrombocytopenia	CAMT (about 91.8%)	(25-30)
Type 2 S505N	Autosomal dominant Germline mutation, rare somatic mutation	Autonomous activate MPL function	Thrombocytosis	HT (about 54.5%)	(22,24,34,35)
K39N,P106L	Autosomal dominant with incomplete penetrance and autosomal recessive with possible mild heterozygote manifestation germline mutation	Reduce binding affinity to ligand thrombopoietin	Thrombocytosis	HT (7% of African-American individuals and 6% of Arab individuals)	(40,41)
Type 3 W515L/K	Somatic mutation	Autonomous activate MPL function	Thrombocytosis	MPNs (about1% in ET and 5% in PMF, rare in PV), AMKL with myelofibrosis (about 25%),rare in RARS-t	(48-50,57,59,66-69)
Type 4 W515A/R, T487A,Y252H	Somatic mutation	Autonomous activate MPL function	Thrombocytosis	Rare in MPNs、 AMKL and ET respectively	(52,57,59,70,71)
A519T,L510P and A506T	Somatic mutation	No function or maybe regulate other genetic events.	Unknown	Rare in PMF	(57)
W515-P518delinsKT, Y591D,S204P/F, IVS 11/12 and IVS 10/11	Somatic mutation	Unclear	Unknown	Rare in MPNs	(72-74)

### MPL mutations found in CAMT

Currently, 41 different *MPL* mutations have been detected in CAMT samples [25]. They can be classified into nonsense mutations, deletions and frameshift mutations, missense mutations and splice site mutations. Ballmaier et al. analysed 9 patients with CAMT and found that the types of *MPL* mutations correlated with the clinical course of the disease [26]. King then proposed a new classification of CAMT, type I and type II, based on the course of the disease and the types of *MPL* mutations [27].

CAMT type I is characterised by persistently low platelet counts and early progression to bone marrow failure. Nonsense, deletions and frameshift *MPL* mutations, such as *MPL* c.127C>T and c.378delT, may be responsible for this disease type, as these mutations introduce premature stop codons and thus cause a complete loss of CD110 function [28]. In *in vitro* assays, CD110 is not expressed in K562 cells with nonsense mutations; therefore, no TPO-driven signalling can be detected [29]. Colony assays have also revealed that the total number of colony forming cells derived from CAMT I patients with nonsense mutations was significantly reduced compared with normal donors [28].

CAMT II displays a less aggressive course with a transient increase in platelet counts during the first year and delayed or no progression of pancytopenia. Additionally, types of missense and splice site *MPL* mutations, such as the most frequent mutation, c.305G>C, are closely associated with CAMT II. This is because these mutations cause amino acid changes or mRNA splicing defects in CD110, which do not completely abrogate the function of CD110 but leave residual activity [28-30]. Two lines of evidence support this hypothesis: 1. TPO-driven signalling is significantly reduced but not eliminated in K562 cells with missense mutations in *in vitro* assays [29] and 2. Colony-forming cells derived from CAMT II patients with missense mutations remain reactive to TPO [28].

In general, several studies have confirmed the genotype-phenotype correlation in CAMT [28,31,32]. However, as few reports discuss the relationship between *MPL* mutations and brain abnormalities, malignant transformation in CAMT is still unclear [23,32,33]. Due to the causative function of *MPL* mutations, especially nonsense mutations, in CAMT, gene therapy aimed to correct CD110 deficiency seems possible.

### *MPL*-S505N, K39N, and P106L mutations associated with HT

*MPL*-S505N is an activating mutation that was first detected by Ding et al. in all members of a specific Japanese family with HT [22]. They found that the Ba/F3 cells transfected with the mutant type (S505N) but not the wild type DNA survived, even without growth factor stimulation [22]. Additionally, a study has demonstrated the constitutive activation of downstream signals without factor dependence in both Ba/F3 cells with the S505N mutation and platelets of the S505N mutant derived from HT patients [22]. How does *MPL*-S505N induce ligand-independent phosphorylation of downstream signalling molecules? At least two mechanisms have been elucidated: 1. *MPL*-S505N transduces the signal via the autonomous dimerisation of the *MPL* protein due to strong amino acid polarity [34] and 2. *MPL*-S505N leads to movement of the transmembrane domain and conformational changes, including tilt and azimuthal rotational angles along the membrane axis, which alter the conformation of the nearby intracellular domain and thus induce constitutive signal activation [35].

To elucidate the biologic profile of MPNs, Teofili et al. have assessed 38 children with PV and ET [36]. However, when they conducted the *MPL* gene mutation analysis, they identified MPL-S505N in 9 of 11 children with ET, thereby suggesting that these paediatric cases of familial ET are in fact HT, which led them to question the revised WHO diagnostic criteria for ET. To evaluate this observation, they increased the number of children in the study and still found erroneous diagnoses for children with ET [37]. Therefore, they proposed that to avoid erroneous diagnoses, the first step of the diagnostic screening should be to test for *MPL*-S505N in those children with thrombocythaemia who had a familial history [38]. Recently, the same group extended their study to include 44 HT patients, including both children and adults, and found that 22 of the 44 patients (approximately 54.5%) carried the *MPL*-S505N mutation. At the median follow-up of 9 years for the 22 patients with this mutation, they observed thrombotic manifestation in 4 cases, myocardial infarction in 7 cases, foetal loss in 2 cases, deep vein thrombosis of the leg and Budd Chiari syndrome in 2 cases, and splenomegaly and bone marrow fibrosis in almost all patients. Therefore, they concluded that *MPL*-S505N was associated with clinical manifestations such as a significant thrombotic risk, which, with age, evolved to splenomegaly and bone marrow fibrosis, thereby noticeably affecting patient life expectancy [24]. Interestingly, most of the HT cases reported with *MPL*-S505N were from Italian families; Liu et al. have also observed this phenomenon, for which they argue that a founder effect in Italian pedigrees with HT may explain this occurrence [39].

*MPL*-K39N is a polymorphism restricted to African Americans, and approximately 7% of African Americans are heterozygous for this mutation [40]. In contrast, *MPL*-P106L was found in approximately 6% of Arabic individuals but not in Caucasian individuals [41]. These observations may indicate ethnic specificity for these two mutations. Both of these mutations are located within the extracellular domain of CD110, which may affect TPO binding, thereby leading to the defective clearance of TPO and thus causing thrombocythaemia. However, whether the two mutations correlate with complications, such as thrombosis or bleeding, requires additional statistical analysis of the data.

### *MPL*-W515L/K associated with MPNs, RARS-t and AML

The two mutations were independently assessed because the most recent 2008 WHO diagnostic criteria for MPNs highlights the function of the *MPL*-W515L/K mutations in the diagnosis of ET and PMF [42]. This was the first time that these mutations appeared in the diagnostic criteria.

Although JAK2V617F is the most common mutation in BCR-ABL-negative MPNs, approximately 50% of ET and PMF patients are JAK2V617F-negative [43-47]. In 2006, *MPL*-W515L was identified by Pikman et al., and *MPL*-W515K was discovered several months later [48,49]. The frequency rates of *MPL*-W515L/K in ET and PMF patients are approximately 1% and 5%, respectively, but they are rarely found in PV patients [48,50]. Interestingly, the proportion of the *MPL*-W515L mutations is higher than that of *MPL*-W515K mutations in MPN patients, although the ratio of homozygous patients is lower for the *MPL*-W515L mutation [51-53]. These mutations were identified not only in granulocytes but also in monocytes, B cells, T cells, NK cells, and even haematopoietic stem cells [54-56]. However, increased mutation levels in the myeloid lineage combined with the observation that MPN is a myeloproliferative rather than a lymphoproliferative disorder suggest a proliferative advantage toward the myeloid series induced by *MPL*-W515 mutations [54].

The following aspects clearly indicate the close relationship between *MPL*-W515L/K mutations and the pathogenesis of MPNs. 1. *MPL*-W515L/K mutations are located in the unique amphipathic KWQFP motif of CD110, which is important for preventing its spontaneous activation [5]. Thus, *MPL*-W515L/K mutations may cause spontaneous activation. In *in vitro* assays, several signalling pathways were constitutively phosphorylated and the S phase of the cell cycle was enhanced in *MPL*-W515L/K-expressing cell lines; even when deprived of cytokines, cytokine-independent colony formation and higher megakaryocytic cloning efficiency have been reported [49,57]. In this process, the cytosolic tyrosine 112 of CD110 *MPL*-W515 mutants is crucial and represents a potential target for pharmacologic inhibition [58]. **2**. In *in vivo* assays, the analysis of tumourigenesis induction capacity of *MPL-*W515L/K mutants in nude mice revealed a clinical phenomenon similar to that observed with ET and PMF and showed atypical megakaryocytic hyperplasia, splenomegaly and thrombocytosis [49,57,59]. **3.** Currently, *MPL*-W515L/K mutations have not been observed in normal individuals, and several studies have indicated that MPN patients with *MPL*-W515L/K mutations present with older age, lower haemoglobin levels and higher platelet counts. However, the relationship between mutation and complications, such as thrombosis, remains unclear [51,53,60]. **4.** Two *MPL*-W515L mutation-positive PMF patients were treated with allogeneic stem cell transplantation (allo)-SCT, and the *MPL*-W515L mutation was not detected after allo-SCT and mutation status remained negative for a long follow-up period, which is in line with the absence of clinical or molecular relapse [61]. This observation suggests that the *MPL*-W515L mutation could be used as a minimal residual disease marker and shows that *MPL*-W515L plays a pathogenic role in MPN. **5.** As mentioned above, *MPL*-W515L/K mutations may cause spontaneous signal transduction, including that of the JAK-STAT/Crkl pathway. Moreover, various small molecule JAK kinase inhibitors not only inhibit spontaneous activation, thereby reducing the colony-forming capacity of cells with *MPL*-W515L/K mutation in *in vitro* assays, but also effectively relieve the symptoms observed in a *MPL*-W515L murine model of MPN [59,62]. Additionally, JAK kinase inhibitors display therapeutic value for MPN patients with *MPL*-W515L/K mutations [63-65]. These observations confirm the status of *MPL*-W515L/K in MPNs.

The *MPL*-W515L mutation has also been identified in RARS-t and AML patient samples. Currently, RARS-t patients harbouring the *MPL*-W515L mutation are rare [66-68]. However, the mutation rate of *MPL*-W515L has been found to be as high as 25% in acute megakaryoblastic leukaemia (AMKL) with myelofibrosis, which is a subtype of AML [69]. The *MPL*-W515L mutation may also be involved in the pathogenesis of RARS-t and AML; however, as reports on this topic are sparse, the function of the mutation in these two diseases remains uncertain.

### Other *MPL* mutations

Due to the extremely low mutation rate and unknown function of some mutations, we classified these mutations together into one group. Compared with the *MPL*-W515L/K mutations, *MPL*-W515A/R mutations were found to be significantly less frequent [48,52,57]. However, it is conceivable that *MPL*-W515A/R mutations function via a mechanism similar to that of the *MPL*-W515L/K mutations, as they are mutations of the same amino acid. *MPL*-A519T, L510P and A506T mutations are not gain-of-function mutations, and some scholars have hypothesised that these mutations have no relevance to haematological disease or may be regulators of other genetic events [57]. In contrast to other mutations, *MPL*-T487A was observed in an acute megakaryoblastic leukaemia (AMKL) patient and induces a myeloproliferative disorder in mice by conferring the ability of cytokine-independent cell growth [70]. The recently detected *MPL-*Y252H mutation in ET also displays a growth-promoting effect [71]. However, the function of the *MPL*-Y591D, W515-P518delinsKT, S204P/F, IVS 11/12 and IVS 10/11 mutations remains unknown [72-74].

## Conclusions

**Figure 2:**
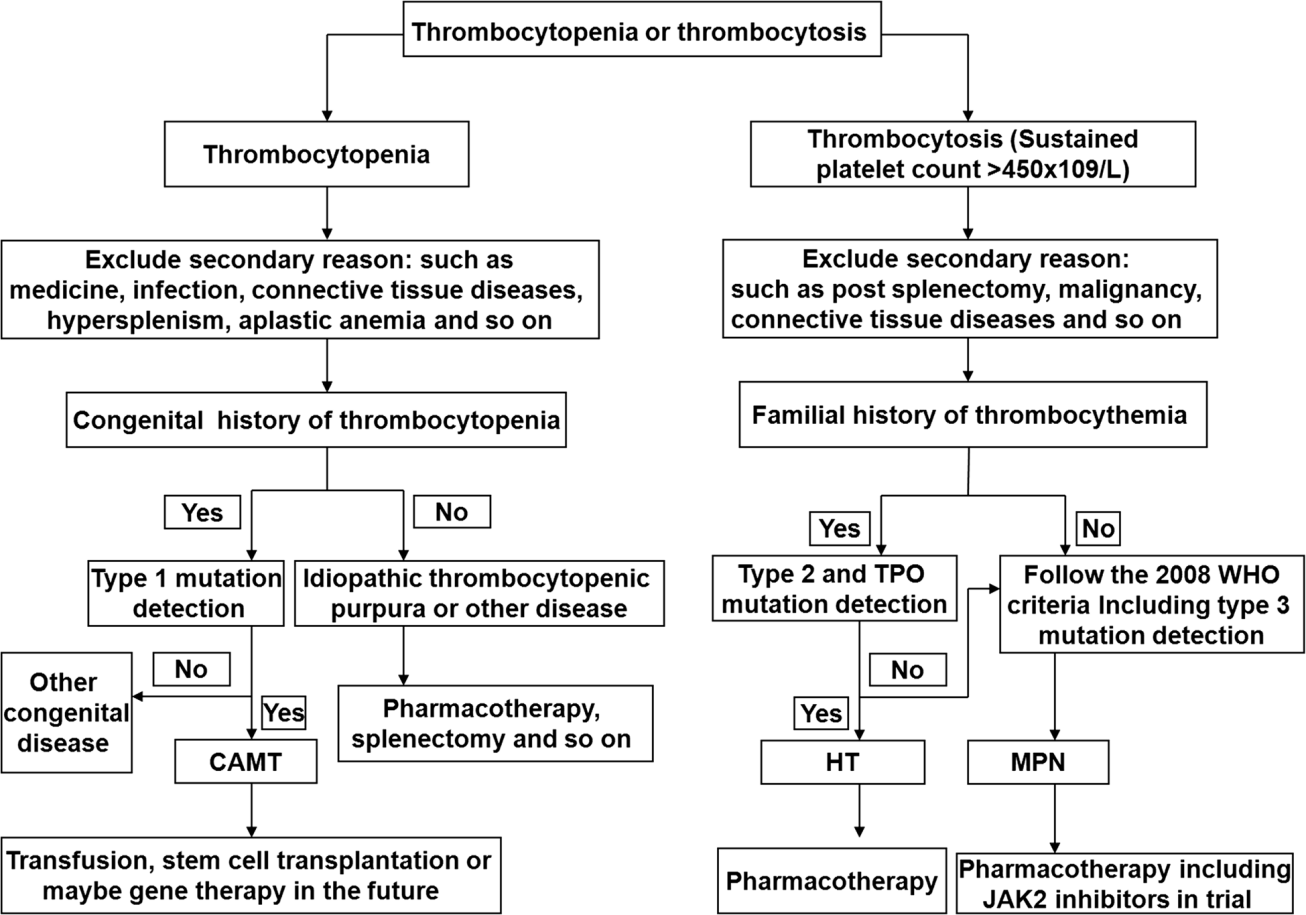
A proposed strategy for management of patients with thrombocytopenia or thrombocytosis.

In conclusion, several *MPL* mutations have been identified in association with haematopoietic diseases. These mutations may alter the normal regulatory mechanisms and lead to autonomous activation or signal deficiency. In this review, we assessed 59 different *MPL* mutations and classified these mutations into four different groups based on the associated diseases and mutation rates. Using this classification, we clearly demonstrate the existence of four diverse types of *MPL* mutations and their clinical significance. Different types of *MPL* mutations induce distinct haematopoietic diseases, and the same haematopoietic diseases may be caused by different mutations. Therefore, individual treatment regimens are required to intervene in haematopoietic disease based on the type of mutation present. We proposed a strategy for patients with thrombocytopenia or thrombocytosis, especially with a family history and congenital history (Summarised in Figure 2). For example, for patients with thrombocytopenia, excluding secondary causes (i.e., medicine, infection, connective tissue diseases, hypersplenism or aplastic anaemia), a screening for Type 1 *MPL* mutations should be performed, especially for patients with a congenital history. If a Type 1 mutation is present, a diagnosis of congenital amegakaryocytic thrombocytopenia can be suggested and an appropriate therapeutic schedule can be formulated, including transfusion, stem cell transplantation or even gene therapy in the future. Otherwise, other congenital diseases should be considered. For patients without a congenital history, primary and exclusive diseases may be considered, such as idiopathic thrombocytopenia purpura or other diseases, and pharmacotherapy, splenectomy and other therapies should be evaluated. When encountering patients with thrombocytosis (Sustained platelet count > 450x10^9^/L), excluding secondary reasons such as post-splenectomy, malignancy, and connective tissue diseases, Type 2 and TPO mutation screenings should be performed if the patients have familial history. If the patients test negative, a Type 3 mutation screening should be carried out. When patients test positive for Type 2 and TPO mutations, an HT diagnosis should be considered. However, when patients test negative for Type 2 and TPO mutations, sporadic thrombocythaemia should be considered while adhering to the 2008 WHO criteria [42]. Perhaps in the near future, when a diagnosis of MPN according to the 2008 WHO criteria is possible, JAK2 inhibitors that are currently being assessed in trials may be available for patients.

Nevertheless, several questions regarding *MPL* mutations remain unresolved. Why does one mutation, such as *MPL-*W515L, give rise to several disease phenotypes, namely ET, PMF, RARS-T and AML? The *MPL*-S505N mutation found in HT samples is a germline mutation; however, three acquired mutation cases were recently reported for MPNs [53,57]. Is this a coincidental phenomenon, and do other *MPL* mutations also possess this property? Finally, do JAK kinase inhibitors also have therapeutic potential for patients with *MPL* mutations, such as *MPL*-S505N, that also cause signal activation?

## Abbreviations

*MPL*: Human c-mpl gene; CAMT: Congenital amegakaryocytic thrombocytopenia; HT: Hereditary thrombocythaemia; MPNs: Myeloproliferative neoplasms; ET: Essential thrombocythaemia; PMF: Primary myelofibrosis; PV: Polycythaemia vera; AMKL: Acute megakaryoblastic leukaemia; RARS-t: Refractory anaemia with ringed sideroblasts associated with marked thrombocytosis.

## Competing interest

The authors declare that they have no conflict of interest relating to the publication of this manuscript.

## Authors’ contributions

YJ and XQ contributed to data preparation. XH, ZC and XZ were involved in concept design, data collection, and manuscript preparation. All authors read and approved the final manuscript.
